# Gas chromatography-mass spectrometry-based untargeted metabolomics reveals metabolic perturbations in medullary thyroid carcinoma

**DOI:** 10.1038/s41598-022-12590-x

**Published:** 2022-05-19

**Authors:** Morteza Ghazanfari Jajin, Raziyeh Abooshahab, Kourosh Hooshmand, Ali Moradi, Seyed Davar Siadat, Roghieh Mirzazadeh, Koorosh Goodarzvand Chegini, Mehdi Hedayati

**Affiliations:** 1 Department of Clinical Biochemistry, School of Medicine, Shahid Sadoughi University of Medical Sciences and Health Services, Yazd, Iran; 2 Cellular and Molecular Endocrine Research Center, Research Institute for Endocrine Sciences, Shahid Beheshti University of Medical Sciences, Tehran, Iran; 3 Curtin Medical School, Curtin University, Bentley, 6102 Australia; 4 Steno Diabetes Center Copenhagen, Gentofte, Denmark; 5 Department of Mycobacteriology and Pulmonary Research, Pasteur Institute of Iran, Tehran, Iran; 6 Microbiology Research Center (MRC), Pasteur Institute of Iran, Tehran, Iran; 7 Department of Biochemistry, Pasteur Institute of Iran, Tehran, Iran

**Keywords:** Hormones, Metabolomics, Biochemistry, Cancer, Cancer metabolism, Cancer screening, Endocrine cancer

## Abstract

Medullary thyroid cancer (MTC) is a rare tumor that arises from parafollicular cells within the thyroid gland. The molecular mechanism underlying MTC has not yet been fully understood. Here, we aimed to perform plasma metabolomics profiling of MTC patients to explore the perturbation of metabolic pathways contributing to MTC tumorigenesis. Plasma samples from 20 MTC patients and 20 healthy subjects were obtained to carry out an untargeted metabolomics by gas chromatography–mass spectrometry. Multivariate and univariate analyses were employed as diagnostic tools via MetaboAnalyst and SIMCA software. A total of 76 features were structurally annotated; among them, 13 metabolites were selected to be differentially expressed in MTC patients compared to controls (*P* < *0.05*). These metabolites were mainly associated with the biosynthesis of unsaturated fatty acids and amino acid metabolisms, mostly leucine, glutamine, and glutamate, tightly responsible for tumor cells' energy production. Moreover, according to the receiver operating characteristic curve analysis, metabolites with the area under the curve (AUC) value up to 0.90, including linoleic acid (AUC = 0.935), linolenic acid (AUC = 0.92), and leucine (AUC = 0.948) could discriminate MTC from healthy individuals. This preliminary work contributes to existing knowledge of MTC metabolism by providing evidence of a distinctive metabolic profile in MTC patients relying on the metabolomics approach.

## Introduction

Medullary thyroid carcinoma (MTC) is a rare malignancy of the neuroendocrine system that originates from calcitonin-secreted cells (C-cells or parafollicular cells) within the thyroid gland. MTC contributes to 5–10% of all thyroid malignancies and roughly 13.4% of deadly thyroid cancers^1,2^. There are two types of MTC, including sporadic MTC (sMTC) and hereditary MTC (hMTC), with an incidence rate of roughly 75% and 25%, respectively^1,3^. The ideal survival of MTC patients mainly depends on the time from diagnosis to apply treatment strategies^4^. Overall, the 10-year survival rate of MTC patients is 90% when all disease is confined to the thyroid and 70% and 20% when cancer has spread to the lymph nodes in the neck and distant areas, respectively^5^.

There are several important diagnostic ways of MTC as follows: ultrasound-guided fine-needle aspiration biopsy (FNAB) with the cytopathological examination, serum calcitonin, and carcinoembryonic antigen (CEA) measurement, and detection of RET germline mutations^6,7^. Despite their safety and efficacy in terms of diagnosis, these approaches suffer from a number of drawbacks. An example of these problems is utilizing FNAB, which sometimes cannot discriminate between benign and malignant lesions; subsequently, 10–30% of cases remain indeterminate^8^. In this regard, to obtain a definitive diagnosis, surgery and nodule removal are necessary^9^. In reference to the published studies, the rate of FNAB with cytological detection for MTC is only 56.4%^6^. Moreover, serum calcitonin level in MTC patients is a sensitive and specific marker; however, an increased in the calcitonin secretion can also be seen in chronic thyroiditis and C cell hyperplasia^10^. Hence, finding an accurate tool and a novel clinical biomarker with high sensitivity and specificity for early detection and management of MTC is critical, which ultimately leads to choosing effective treatments.

Metabolomics accompanied by chemometric analysis is one of the most potent “omics” techniques, primarily used in cancer biology to systematically determine altered metabolites undergoing tumorigenesis in a biological sample^11^. Numerous cancer studies have shown that the metabolomics approach was helpful in early diagnosis and following disease progression in other tissues and organs^12–16^. Furthermore, several metabolomics studies have investigated the alteration of metabolites associated with thyroid cancers^17–20^; however, no metabolomics studies have been performed in MTC patients so far. From this perspective, understanding perturbation in metabolic pathways of MTC by using metabolomics-based techniques can be effective in identifying critical metabolites with potential diagnostic and innovative therapeutic significance.

Metabolomics performed by mass spectrometry (MS) coupled with gas chromatography (GC) provides high sensitivity for identifying and quantifying metabolites and reduces the complexity of metabolite separation^21–23^. Indeed, due to its electron impact (EI) ionization and reproducibility, GC–MS has been recognized as a gold standard in metabolomics-based technique for the comprehensive analysis of metabolites^24–26^. With that in mind, this study intends to determine perturbation in MTC patients’ metabolic pathways compared to healthy subjects by performing GC–MS-based untargeted metabolomics.

## Materials and methods

### Study design

This case–control study was performed on patients referred to the Cellular and Molecular Endocrine Research Center, Research Institute for Endocrine Sciences, Shahid Beheshti University of Medical Sciences, Tehran, Iran. Blood was taken from the case population, including 20 MTC patients (12 females and 8 males) with mean age 45.75 ± 13.841 years. Diagnosis of MTC was based on pathological evidence and clinical outcomes. Moreover, 20 healthy subjects (13 females and 7 males) with mean age 38.25 ± 13.21 were enrolled in the study as a control group. Those using drugs affecting thyroid function or other varieties of cancer and metabolic diseases [metabolic syndrome, diabetes, and insulin resistance (IR)] were ruled out. All participants and healthy volunteers signed written informed consent.

About 10 ml of blood was drawn in anticoagulant ethylenediaminetetraacetic acid (EDTA) ‐containing tubes. Before metabolomics analysis, plasma was separated immediately through blood centrifuging and kept frozen at − 80 °C before metabolomics analysis. The current study followed the principles of the Declaration of Helsinki (1975) and local regulations and was also approved by the local Ethics Committee of Shahid Sadoughi University of Medical Sciences, Medical School, Yazd, Iran (IR.SSU.MEDICINE.REC.1400.130).

### Plasma sample preparation prior to GC–MS analysis

Plasma metabolites were extracted and derivatized using our previously reported article with a minor modification^17^. Briefly, 50 μl plasma of each case was extracted with 1 ml of protein precipitant (methanol/water/isopropanol, 5:2:2, v/v/v). The tubes of mixtures were vortexed for the 60_S_ and chilled at − 20 °C for 20 min, then centrifuged at 14,000 rpm for 15 min at 4 °C. After centrifugation, clear supernatant of each sample was collected and evaporated to complete dryness utilizing Eppendorf vacuum centrifuge for 4 h at 45 °C.

All dried samples were derivatized before injection underwent two-step processes consisting of methoximation followed by trimethylsilylation (TMS). Methoximation was performed by adding 30 μl of methoxyamine hydrochloride in a 20 mg/ml pyridine and mixing for the 30_S_; then, the mixtures were placed on a thermo-shaker at 900 rpm for 1 h at 60 °C. This was accompanied by adding 60 μl of N-Methyl-N-(trimethylsilyl) trifluoroacetamide (MSTFA) as a silylating agent, then samples were mixed and placed on a thermo-shaker to react at 900 rpm for 20 min at 45 °C.

### GC–MS analysis

Gas chromatography–quadrupole mass spectrometry (GC–qMS) analysis was conducted on Agilent 5975C MSD/Agilent 7890A GC system equipped with an HP-5 ms capillary column (Agilent J&W, 30 m × 0.25 μm × 0.25 mm). 1 µl of each derivatized extract sample was injected at a split ratio of 4:1 using helium as a carrier gas with a 1 ml/min flow rate. The inlet, the MS transfer line, and the quadrupole temperatures were set at 280, 230, and 150 °C, respectively. All samples were operated in a randomized order. The initial oven temperature was held at 60 °C for 1 min and then ramped at a rate of 10 °C/min to a final temperature of 280 °C with 10 min hold time. The post-run was 5 min to allow the oven to cool down to 60 °C. The electron ionization (EI) source was set at 70 eV. The GC-qMS data acquisition with full-scan spectra (50–600 *m*/*z*) was recorded in 37.5 min after 5.4 min solvent delay.

### Raw GC–MS data processing

ChemStation Data Analysis software (Agilent Technologies, Palo Alto, CA, USA) was used to transform raw data into CDF format (NetCDF) and then converted to “abf” format by Reifycs Abf converter. Deconvolution, peak identification, gap filling, and peak alignment of metabolites were processed and analyzed automatically using MS-DIAL software with in-built MS/MS reference libraries (v4.60)^27^. The original dataset consisted of the average retention time (RT), the mass-to-charge ratio (*m*/*z*), InChIKey, and peak intensity features exported from MS-Dial for further analysis. Besides, the NIST Mass Spectral Search Program (version 2.0) was applied to confirm all metabolite spectra recognized in MS-DIAL against the reference spectrum from the replib, mainlib, and fiehn libraries with a ≥ 70% similarity index. Subsequently, the metabolite peaks were normalized by performing mTIC method^28^.

### Statistical analysis

Statistical significance analysis between healthy versus MTC patients was done using multivariate and univariate analysis. Cubic root transformation and Pareto scaling were performed as transformation and scaling for all selected metabolites using MetaboAnalyst (v5.0, http://www.metaboanalyst.ca). SIMCA-P 14.0 software (Umetrics, Umeå, Sweden) was performed to create the multivariate statistic plots such as orthogonal partial least squares-discriminant analysis (OPLS-DA) to ascertain the extent of differences between experimental groups. Cross-validated predictive residuals (CV-ANOVA)^29^ and permutation tests were used to test the models' reliability. Variable importance in the projection (VIP) score values equals/higher than one were considered important to the model for discrimination. The p-values were adjusted using false discovery rate (FDR) calculations for multiple-testing issues. Volcano plot based on fold-change values, FDR, and the threshold of significance was performed to explore which metabolites annotated in the GC-qMS dataset were the most significant in the test using MetaboAnalyst, (v5.0). Heatmap, box-and-whisker plots, and receiver operating characteristic (ROC curve) analysis were applied using multiple experiment viewer (MeV) and MetaboAnalyst (v5.0). Potentially identified plasma metabolites were used to investigate the main biological pathways among MTC patients. In this regard, the enrichment-pathway analysis was conducted utilizing the MetaboAnalyst (v5.0) with thresholds of *p* value < 0.05 and FDR < 0.1 as significant.

## Results

### Clinical characteristics of the subjects

The clinicopathological characteristics of subjects are presented in Table 1. This analysis involved 40 participants divided into two groups: MTC (n: 20) and healthy (n: 20). There is no significant difference in mean age between MTC and healthy groups (*P* > 0.05).

**Table 1:** Demographic and clinicopathological characteristics of the study subjects.

Parameter	MTC (n = 20)	Healthy (n = 20)
**Gender**		
Male	8	7
Female	12	13
**Age** (Mean ± SD; years)	45.75 ± 13.841	38.25 ± 13.21
**Tumor size** (Mean ± SD; cm)	2.62 ± 1.20	–
**Capsular invasion**		
Negative	15	–
Positive	5	–
**Vascular invasion**		
Negative	15	–
Positive	5	–
**Perineural invasion**		
Negative	15	–
Positive	5	–
**Lymph node metastasis**		
Negative	13	–
Positive	7	–

*MTC* medullary thyroid cancer.

### Plasma metabolic profile between two groups

Data processing by MS-DIAL yielded 538 compounds, of which 76 metabolites had reliability for further analysis. Metabolites were classified into superclass and class levels based on the ClassyFire system^30^. At the superclass level, metabolites were grouped: 53.33% organic acids and derivatives, 21.33% lipids and lipid-like molecules, 13.33% organic oxygen compounds, 8% organoheterocyclic compounds, and 1.33% homogeneous non-metal compounds. In addition, the plentiful of identified compounds at the class level were ordered as follows: 41.33 carboxylic acids and derivatives, 13.33% organooxygen compounds, and 9.33% hydroxy acids and derivatives (Figure S1).

**Figure 1:**
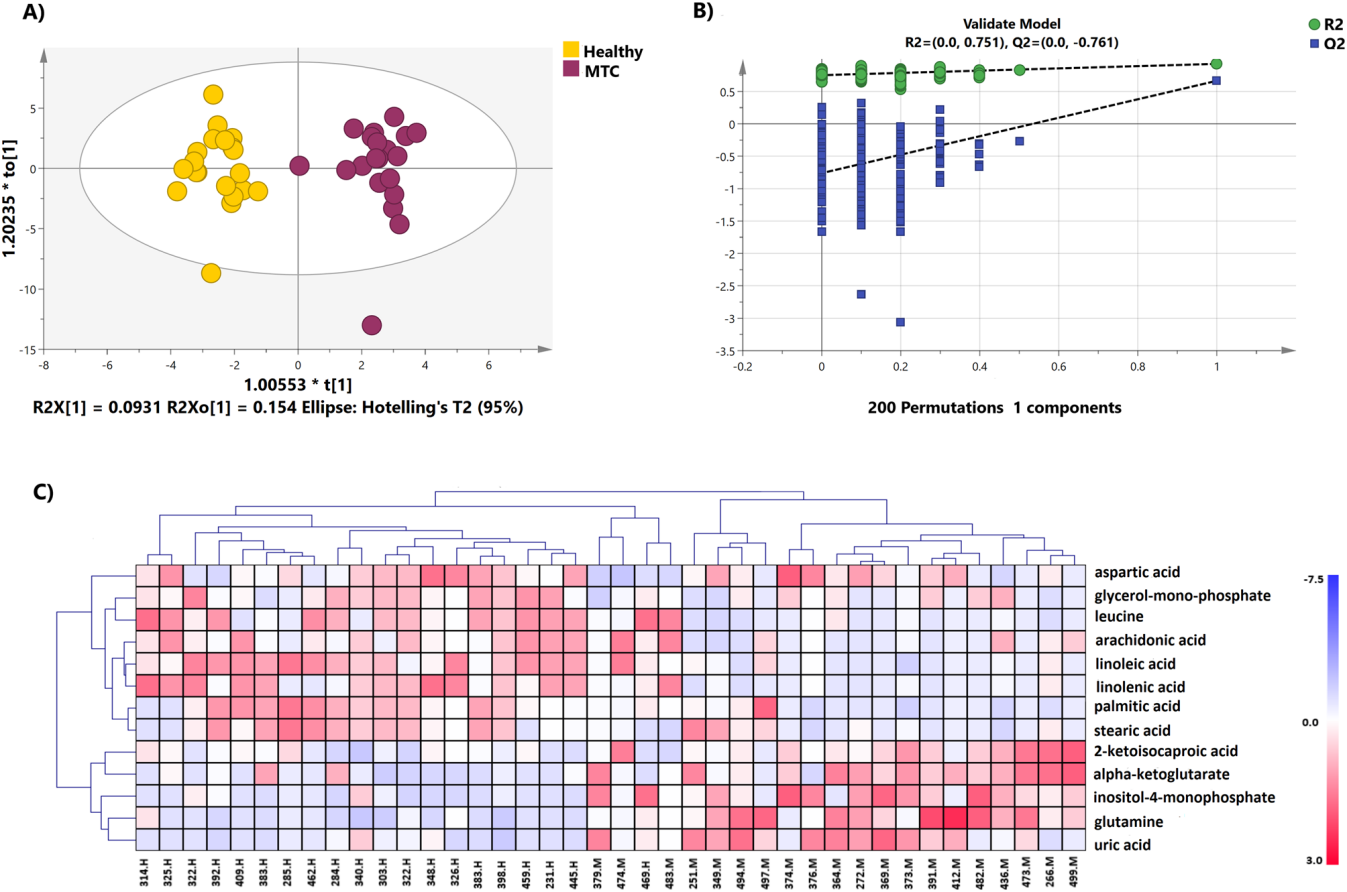
(**A**) OPLS-DA analysis score scatter plot obtained from GC–MS metabolic profiles of the MTC (red dots) and healthy (yellow dots). (**B**) Permutation test with 200 permutations of the OPLS-DA model, showing the model is significant. (**C**) Heatmap visualization of the most significant metabolites with hierarchical clustering analysis (HCA).

A supervised OPLS-DA model score plot showed a perfect separation between MTC and healthy groups (R2X = 0.412, R2Y = 0.925, Q2 = 0.664; Fig. 1A; Table S1). The CV-ANOVA (*p* values < 0.05; Table S1) and permutation tests revealed that the model was significant (Fig. 1B).

Using t-test analysis (*p* values < 0.05, FDR *q* < 0.05) along with a multivariate test with a VIP score of ≥ 1 indicated that there were 13 metabolites out of 76 that differed substantially between the two sets of groups (Table 2). From these data, metabolites that belong to the lipid metabolism, including arachidonic acid, linoleic acid, linolenic acid, palmitic acid, and stearic acid, were reduced in plasma samples of patients with MTC compared to the healthy subjects. The levels of metabolites belonging to the amino acid metabolism, including leucine, glutamine, and aspartate, were considerably altered between the two groups. Besides, other metabolites such as α-ketoglutarate (a keto acid), 2-ketoisocaproic acid (an intermediate of leucine metabolism), glycerol-mono-phosphate (a phosphoric ester of glycerol), and uric acid (generated from the metabolism of purines) were considerably changed in MTC patients compared to the healthy control. The hierarchical clustering heatmap of the 13 significant peak intensities of metabolites between two groups was depicted in Fig. 1C.

**Table 2:** Substantially altered metabolites between MTC and healthy groups using *t* test analysis.

Metabolites	RT (min)	VIP	*P* value	FDR
Linoleic acid	21.14	1.95	2.49E−08	1.90E−06
Leucine	19.15	2.00	1.24E−07	4.70E−06
Linolenic acid	22.66	1.94	2.03E−07	5.13E−06
Palmitic acid	19.61	1.67	1.86E−05	0.000354
Glutamine	17.008	1.66	3.53E−05	0.00046
Glycerol-mono-phosphate	16.59	1.63	3.63E−05	0.00046
Inositol-4-monophosphate	23.26	1.58	0.000159	0.001727
Uric acid	20.36	1.45	0.000712	0.006765
Arachidonic acid	22.49	1.45	0.001053	0.008895
Stearic acid	21.39	1.26	0.002002	0.015214
Aspartic acid	14.12	1.18	0.006998	0.044165
Alpha-ketoglutarate	14.75	1.23	0.007452	0.044165
2-Ketoisocaproic acid	9.97	1.24	0.007555	0.044165

*FDR* false discovery rate, *RT* retention time, *VIP* variable importance in the projection.

*P* values are from the student *t* test which considered < 0.05 statistically significant.

### Most significantly altered metabolites of plasma samples between two groups

**Figure 2:**
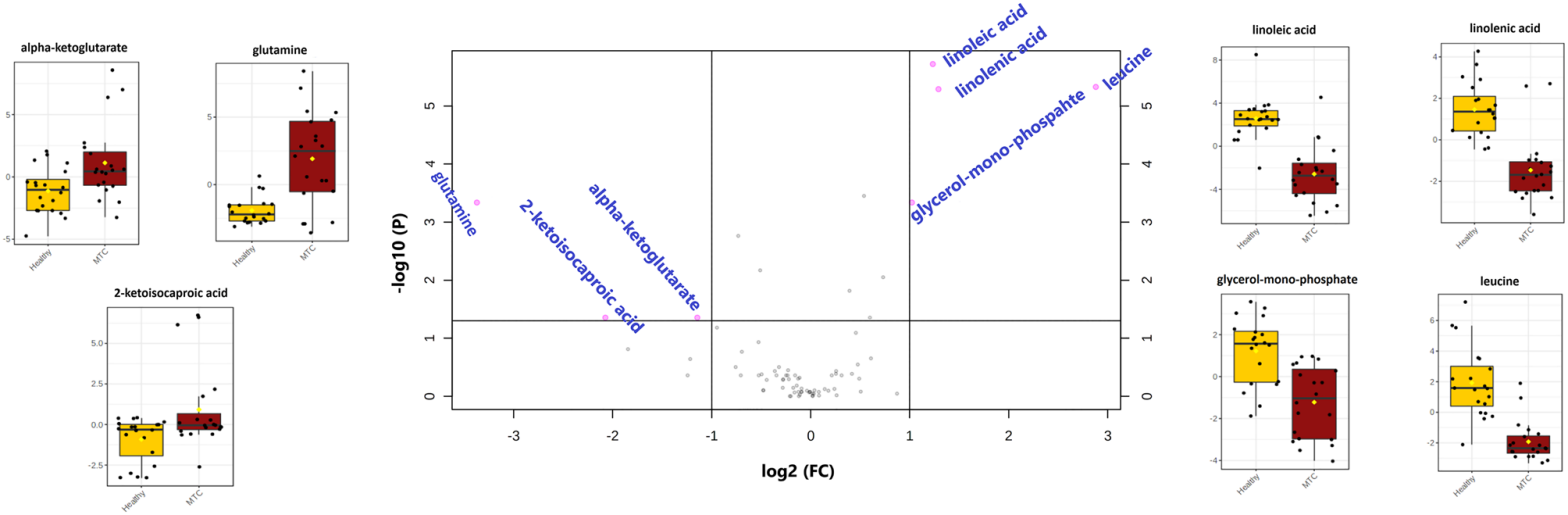
Volcano plot analysis of the most significant metabolite changes comparing MTC versus Healthy. Pink dots on the left indicate metabolites in plasma samples of MTC patients have significantly higher intensity in compression with the healthy group; meanwhile, the pink dots on the right indicate metabolites here are significantly lower in plasma samples of MTC patients compared to healthy subjects. Gray dots refer to all the other identified metabolites with no significant changes between MTC patients and the healthy group. Box and Whisker plots show the normalized values of seven selected metabolites by volcano plot. The *x*-axis shows the specific metabolite, and the *y*-axis is the normalized peak intensity.

To recognize the most significant metabolites differences between the two groups (MTC vs. healthy), univariate volcano plots were applied based on a log2 fold change, the *p* value of < 0.05 and FDR *q* < 0.05. Volcano plot from MTC vs. healthy (Fig. 2) displayed seven metabolites out of 76 remarkably altered, including leucine, α-ketoglutarate, 2-ketoisocaproic acid, glutamine, glycerol-mono-phosphate, linoleic acid, and linolenic acid. The changes in peak intensities of the most prominent metabolites between the two groups were visualized using box plots (Fig. 2).

**Figure 3:**
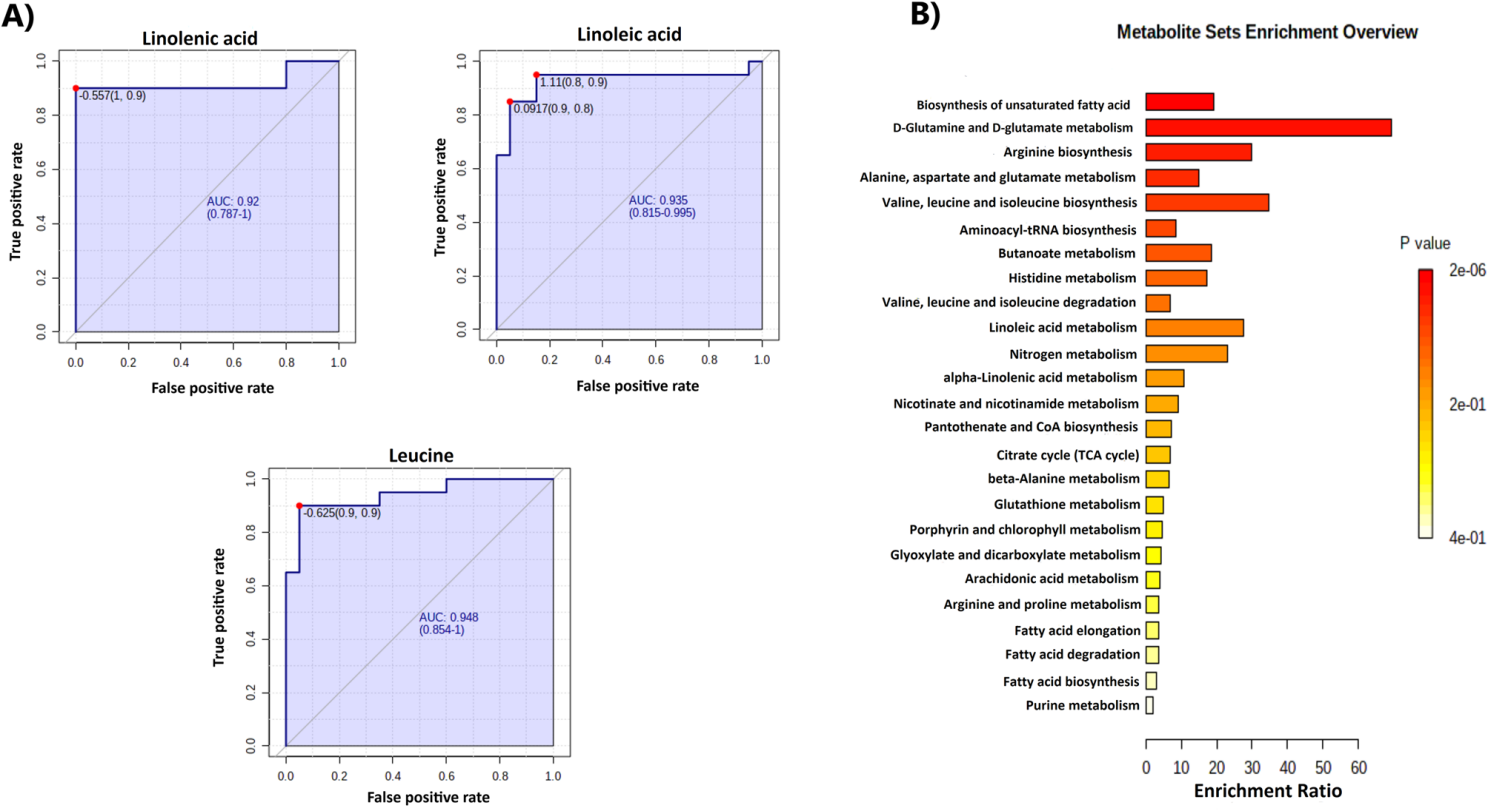
(**A**) ROC curve analyses of the three metabolites' ability to distinguish MTC patients from healthy subjects. (**B**) Metabolic Set Enrichment Analysis (MSEA) indicating top 25 metabolic pathways perturbed upon MTC.

The ROC curve analysis was performed to validate the diagnostic performance of the selected metabolites from volcano plots. The results indicated that the area under the curve (AUC) of 3 metabolites in MTC versus healthy (Fig. 3A) was more prominent than 0.92.

### Pathway analysis

The 13 considerable metabolites were subjected to enrichment analysis utilizing MetaboAnalyst 5.0. The results revealed that these metabolic alterations were mainly associated with biosynthesis of unsaturated fatty acids and biosynthesis and metabolisms of amino acids, mostly glutamine and glutamate metabolism (Fig. 3B). A detailed enrichment analysis table including all the recognized enriched pathways was collected in Table S2.

## Discussion

Currently, cytological evaluation of FNAB thyroid lesions is a standard method for diagnosing MTC ^6,7^. Unfortunately, up to 30% of FNAB results are indeterminate (8), leading to repeat FNAB or tumor surgery for a definitive diagnosis. In recent decades, researchers have sought comprehensive biomarkers to help make a conclusive diagnosis before surgery. Accordingly, several significant biological markers have been reported, including miR-127, miR-154, miR-183, miR-9, miR-224, miR-375, miR-223^31^ calcitonin, CEA, and mutations in RET proto-oncogenes^7^. However, due to their low sensitivity or specificity and unpredictable positive values, it is crucial to find sensitive and specific markers for the early diagnosis of MTC. Researchers have considered metabolomics in the past 2 decades, providing a comprehensive approach with the importance of valid diagnosis by identifying altered metabolites in cancer patients.

**Figure 4:**
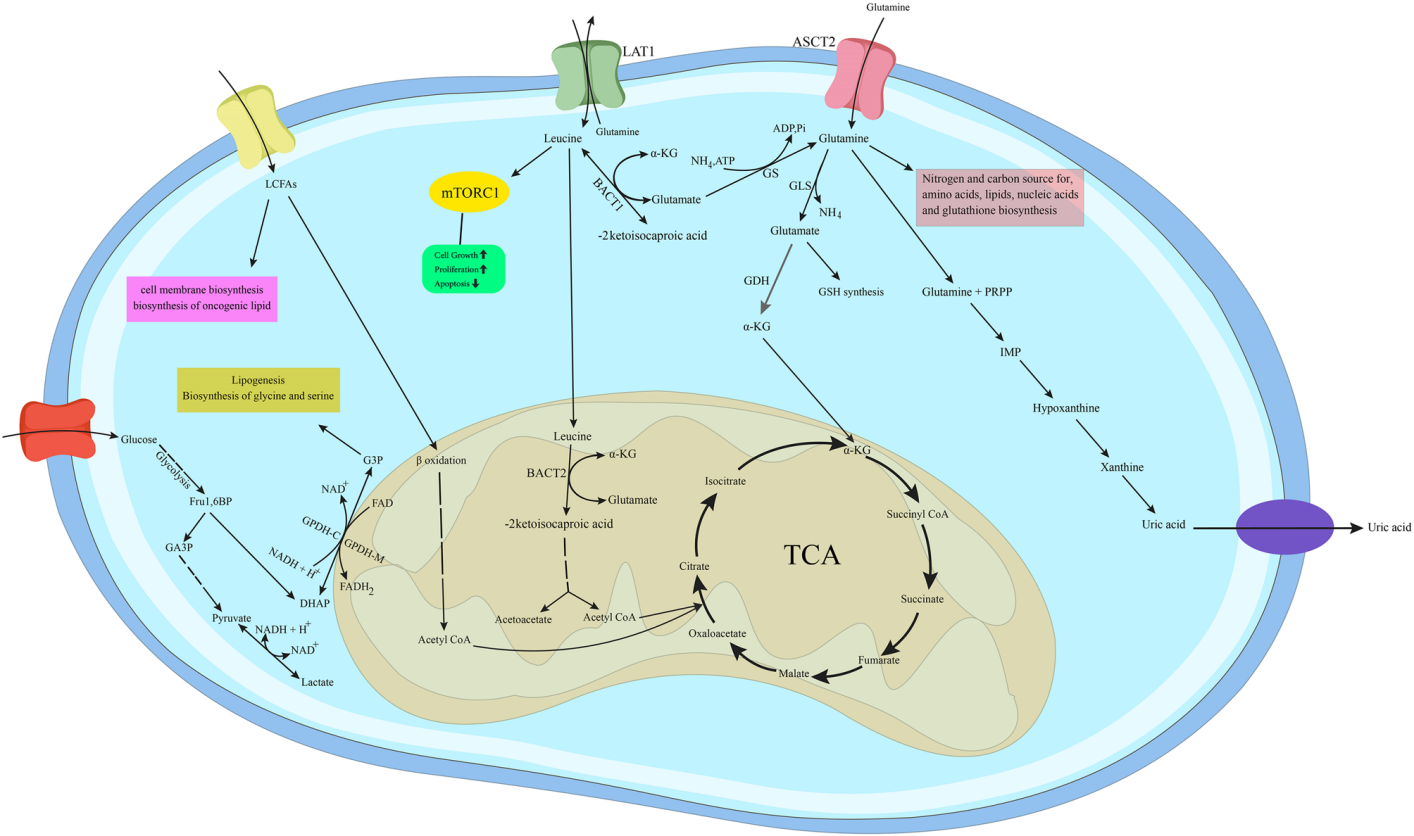
Metabolic reprogramming in MTC. The most crucial metabolic reprogramming in MTC tumorigenesis are leucine and glutamine metabolism, purine catabolism, and G3P shuttle. *TCA* tricarboxylic acid, *α-KG* α-ketoglutarate, *LAT1/SLC7A5* L-type AA transporter, *ASCT2/SLC1A5* alanine-serine-cysteine transporter2, *BCAT1* cytosolic branched-chain aminotransferase, *BCAT2* mitochondrial branched-chain aminotransferase, *mTORC1* mammalian targets rapamycin complex 1, *GS* Glutamine synthetase, *GDH* glutamate dehydrogenase, *ALT* alanine transaminase, *AST* aspartate transaminase, *GLS* glutaminase, *LCFAs* long-chain fatty acids, *G3P* glycerol-3-phosphate, *DHAP* dihydroxyacetone phosphate, *GSH* glutathione, *PRPP* phosphoribosyl pyrophosphate, *IMP* inosine monophosphate.

Several studies on metabolomics-based techniques for detecting thyroid cancer metabolism have been reported; however, none have studied MTC. Therefore, to the best of our knowledge, our work is the first metabolomics study to identify the plasma metabolic profile of MTC patients utilizing the GC–MS method. Our data showed that the perturbation in the metabolism of MTC patients compared to healthy individuals was mainly related to amino acid metabolism, tricarboxylic acid cycle (TCA), fatty acid metabolism, and purine metabolism. These alterations are depicted in Fig. 4.

Changes in the amino acids metabolism and their derivatives in the plasma of patients with MTC compared to healthy individuals were as follows: a decrease in leucine and aspartate and an increase in glutamine, 2-ketoisocaproic acid, and α-ketoglutarate (α-KG). However, the most noticeable change in amino acids, which can differentiate between healthy individuals and MTC patients, was leucine, which is significantly reduced. Leucine is classified as a branched-chain amino acid (BCAAs) that is absorbed by cells through the L-type AA transporter 1 (LAT1/SLC7A5)^32,33^. BCAAs are a carbon and nitrogen source in protein synthesis which oxidized by tumors for energy purposes^34^. In accordance with the present result, previous studies on the other types of thyroid cancers demonstrated depletion in leucine levels. Lu et al.^35^ reported a decrease in the levels of several amino acids, including leucine, in a study of the plasma metabolic profile of patients with papillary thyroid microcarcinoma (PTMC). Moreover, an increase in leucine has also been reported in another study performed on thyroid cancer tissue, indicating an increase in uptake of it by tumor cells^36^. The first stage of leucine catabolism is its transamination to 2-ketoisocaproic acid by the branched-chain aminotransferase (cytosolic BCAT1 or mitochondrial BCAT2), which leads to the glutamate production by transferring the amine group to α-KG^37^. As a consequence, decreased leucine along with increased 2-ketoisocaproic acid and α-KG indicates increased leucine catabolism in MTC patients. Furthermore, leucine plays a role in nutrient signaling, activating the mammalian target of rapamycin (mTOR)^38,39^. Several studies showed that the AKT/mTOR pathway in the MTC is highly triggered. Activating this pathway is essential since it promotes tumor cells' growth, proliferation, and survival^40–42^. Therefore, leucine could be a major factor, if not the only one, causing MTC tumorigenesis by affecting signaling pathways which could be an interesting marker for further study in this area.

Glutamine is a non-essential amino acid (NEAA) that enters the cell via the alanine-serine-cysteine transporter 2 (ASCT2/SLC1A5)^33,43^ and was another prominently increased amino acid in the current study. Our finding was in agreement with a previous study by Zhou et al.^20^, who reported an increase in glutamine of PTC patients serum compared to healthy individuals but differed from Huang et al.^44^, who found a decrease in glutamine of PTC patients. Glutamine plays a vital role in metabolism and is the primary nitrogen and carbon source for amino acids, lipids, nucleic acids, and glutathione biosynthesis^34,45^. Tumor growth under hypoxic conditions or mitochondrial dysfunction depends almost exclusively on the metabolism of glutamine^46^. Glutaminolysis is a process that begins with glutamine turning to glutamate, catalyzed by glutaminase (GLS)^47^. Glutamic acid can be further converted to α-KG by oxidative deamination with glutamate dehydrogenase (GDH) or transaminase^34^. In addition, glutamate can be converted to other amino acids, including alanine and aspartate, by the action of alanine transaminase (ALT) and aspartate transaminase (AST), respectively. In a study by Kim et al. examining the expression of proteins involved in glutamine metabolism in thyroid cancer, they found that ASCT2 expression was increased in MTC tumors^48^. ASCT2 has been suggested to play a central role in glutamine metabolism and maintaining tumor growth^33^. ASCT2 increases the intracellular concentration of glutamine, which in turn promotes the uptake of essential amino acids, especially leucine, into the cell by activating glutamine flux outwards by LAT1, and activates mTORC1^33,49^. This could be a reason for the increment of glutamine in the plasma of MTC patients. Hence, a relative decrease in plasma levels of glutamate and drastically increase in α-KG, and glutamine suggest an overall increase in glutaminolysis process and mitochondrial dysfunction in MTC patients.

In the present results, another noticeable perturbation was related to lipids metabolism. We observed a considerable decrease in long-chain fatty acids (LCFAs) in MTC patients compared to healthy individuals, including palmitic acid (16:0), stearic acid (18:0), linoleic acid (18:2), linolenic acid (18:3), and arachidonic acid (20:4). The results were predictable because the significant differences in the metabolism of cancer cells and normal cells are linked to the pathways involved in energy. Free fatty acids are essential for energy, especially when glucose levels are insufficient. Beta-oxidation of fatty acids provides the energy needed for tumor cells' growth and proliferation^50^. Also, another cause of low levels of lipids and fatty acids in the plasma of MTC patients can be associated with increased demand for lipids for tumor cell membrane biosynthesis, which leads to increased use of fatty acids. Linoleic acid and linolenic acid are precursors to arachidonic acid, and arachidonic acid is a precursor to prostaglandins, which are oncogenic lipid signaling molecules^11,50^. It can thus be assumed that perturbed lipid metabolism and subsequently increased oncogenic lipid signaling play a critical role in MTC tumorigenesis.

Another result from our study is the higher uric acid levels in MTC compared to healthy subjects, which is the end product of purine catabolism. It seems that increased cell growth and proliferation in MTC patients are associated with increased demand for nucleotides, which increases purine catabolism and subsequently increases uric acid levels in these patients.

An interesting finding from our study was decreased glycerol-mono-phosphate levels in MTC patients. The main form of glycerol-mono-phosphate in plasma is glycerol-3-phosphate (G3P). According to Otto Warburg, glucose oxidation in cancer cells does not lead to oxidative phosphorylation in mitochondria but instead leads to excess NADH and lactate production^51^. The G3P shuttle is a mechanism for NADH reproduction from NAD + in the human body. In this shuttle, the cytoplasmic enzyme G3P dehydrogenase 1 (GPDH-C) catalyzes the conversion of dihydroxyacetone phosphate to G3P^52,53^. Indeed, G3P is defined as a metabolite linking glycolysis, lipogenesis, and oxidative phosphorylation (OXPHOS)^54^. Therefore, it can be assumed that the decrease in glycerol-mono-phosphate plasma levels is due to increased biosynthetic processes in MTC patients.

## Conclusion

All in all, in this preliminary study, we identified the metabolic characteristics of MTC patients and healthy individuals using mass spectrometry-based metabolomics approach for the first time, which showed significant similarities and differences in specific metabolic pathways. All MTC samples indicated changes in amino acid metabolism, fatty acid metabolism, purine metabolism, and TCA intermediates, suggesting that MTC has altered metabolic pathways compared to the healthy group due to increased energy requirements and macromolecular precursors for the synthesis of proteins and lipids. According to the results of 13 metabolites whose changes were significant in the MTC and healthy group, three metabolites, including linoleic acid, linolenic acid, and leucine, can be used as potential tumor biomarkers for early detection of medullary thyroid cancer. Future studies which take these variables into account need to be undertaken.

## Supplementary Information

Supplementary Information.

## Data Availability

All data that support all the experimental findings in this article is available in the Supplementary Data File provided.
