# Supplementary material for: Gas chromatography-mass spectrometry-based untargeted metabolomics reveals metabolic perturbations in medullary thyroid carcinoma
Source: Sci Rep. 2022 May 19;12:8397. doi: 10.1038/s41598-022-12590-x (PMC9120505; doi:10.1038/s41598-022-12590-x)
Supplement: Supplementary file 1 — Supplementary Information. [file 41598_2022_12590_MOESM1_ESM.docx]

**Gas chromatography-mass spectrometry-based untargeted metabolomics reveals metabolic perturbations in medullary thyroid carcinoma**

Morteza Ghazanfari Jajin^1^, Raziyeh Abooshahab^2,3^, Kourosh Hooshmand^4^, Ali Moradi^1^, Seyed Davar Siadat^5,6^ Roghieh Mirzazadeh^7^, Koorosh Goodarzvand Chegini^1*^, Mehdi Hedayati^2*^

^1^Department of Clinical Biochemistry, School of Medicine, Shahid Sadoughi University of Medical Sciences and Health Services, Yazd, Iran.

^2^Cellular and Molecular Endocrine Research Center, Research Institute for Endocrine Sciences, Shahid Beheshti University of Medical Sciences, Tehran, Iran

^3^Curtin Medical School, Curtin University, Bentley 6102, Australia

^4^Steno Diabetes Center Copenhagen, Gentofte, Denmark

^5^Department of Mycobacteriology and Pulmonary Research, Pasteur Institute of Iran, Tehran, Iran

^6^Microbiology Research Center (MRC), Pasteur Institute of Iran, Tehran, Iran

^7^Department of Biochemistry, Pasteur Institute of Iran, Tehran, Iran


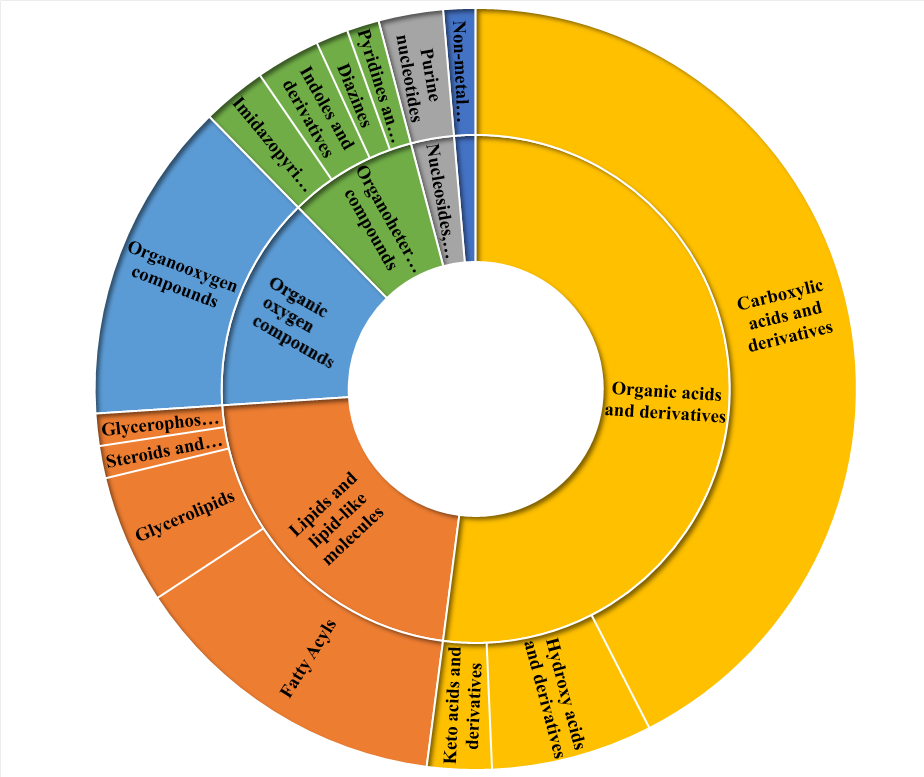


**Figure S1** Annotated human metabolite classes present in plasma samples using the ClassyFire annotation system.

**Table S1** OPLS-DA models’ parameters for plasma samples between MTC and healthy subjects

|  | OPLS-DA Model |
| --- | --- |
| N^a^ | 1 (*p)^d^* +3 (*O)^e^* |
| R^2^X(cum)^b^ | 0.412 |
| R^2^Y(cum)^b^ | 0.925 |
| Q^2^(cum)^c^ | 0.664 |
| CV-ANOVA (p-value) | 1.29E-05 |

^a^ N: number of components.
^b^ R2X (cum) and R2Y (cum) are the cumulative modeled variations in the X and Y matrix, respectively.

^c^ Q2Y (cum) is the cumulative predicted variation in the Y matrix.
^d^ P: Predictive component.
^e^ O: Orthogonal component.

**Table S2** Pathway Enrichment analysis of altered metabolites

|  | ^a^Total Cmpd | ^b^Hits | ^c^Raw p | ^d^FDR |
| --- | --- | --- | --- | --- |
| Biosynthesis of unsaturated fatty acids | 36 | 5 | 2.32E-06 | 0.000195 |
| Glutamine and glutamate metabolism | 6 | 3 | 5.56E-06 | 0.000234 |
| Arginine biosynthesis | 14 | 3 | 9.80E-05 | 0.00275 |
| Alanine, aspartate and glutamate metabolism | 28 | 3 | 0.000835 | 0.0175 |
| Valine, leucine and isoleucine biosynthesis | 8 | 2 | 0.0013 | 0.0218 |
| Aminoacyl-tRNA biosynthesis | 48 | 3 | 0.00407 | 0.0566 |
| Butanoate metabolism | 15 | 2 | 0.00474 | 0.0566 |
| Histidine metabolism | 16 | 2 | 0.00539 | 0.0566 |
| Valine, leucine and isoleucine degradation | 40 | 2 | 0.0319 | 0.298 |
| Linoleic acid metabolism | 5 | 1 | 0.0357 | 0.3 |
| Nitrogen metabolism | 6 | 1 | 0.0427 | 0.326 |
| alpha-Linolenic acid metabolism | 13 | 1 | 0.0903 | 0.632 |
| Nicotinate and nicotinamide metabolism | 15 | 1 | 0.104 | 0.669 |
| Pantothenate and CoA biosynthesis | 19 | 1 | 0.129 | 0.746 |
| Citrate cycle (TCA cycle) | 20 | 1 | 0.136 | 0.746 |
| beta-Alanine metabolism | 21 | 1 | 0.142 | 0.746 |
| Glutathione metabolism | 28 | 1 | 0.185 | 0.91 |
| Porphyrin and chlorophyll metabolism | 30 | 1 | 0.197 | 0.91 |
| Glyoxylate and dicarboxylate metabolism | 32 | 1 | 0.209 | 0.91 |
| Arachidonic acid metabolism | 36 | 1 | 0.232 | 0.91 |
| Arginine and proline metabolism | 38 | 1 | 0.244 | 0.91 |
| Fatty acid elongation | 39 | 1 | 0.249 | 0.91 |
| Fatty acid degradation | 39 | 1 | 0.249 | 0.91 |
| Fatty acid biosynthesis | 47 | 1 | 0.293 | 1 |
| Purine metabolism | 65 | 1 | 0.382 | 1 |

^a^ Total Cmpd: total number of compounds in the pathway

^b^ Hit: actually matched number from the data

^c^ Raw p : p value calculated from the enrichment analysis

^d^ FDR: p value adjusted using False Discovery Rate
